# Redesigning continuing professional development: Harnessing design thinking to go from needs assessment to mandate

**DOI:** 10.1007/s40037-020-00604-1

**Published:** 2020-08-12

**Authors:** Alexander Chorley, Khalid Azzam, Teresa M. Chan

**Affiliations:** 1 Division of Emergency Medicine, Department of Medicine, Faculty of Health Sciences, McMaster University, Hamilton, ON Canada; 2 Hamilton Health Sciences Centre, Hamilton, ON Canada; 3 Division of General Internal Medicine, Department of Medicine, Faculty of Health Sciences, McMaster University, Hamilton, ON Canada; 4 Office of Continuing Professional Development, Faculty of Health Sciences, McMaster University, Hamilton, ON Canada

**Keywords:** Design thinking, Continuing medical education, Continuing professional development, Strategic planning

## Abstract

**Background:**

The world of medicine is constantly changing, and with it the continuing professional development (CPD) needs of physicians. As the CPD landscape is shifting away from unidirectional delivery of knowledge through live large group learning (conferences) and is placing increased emphasis on new approaches for skills training not taught a decade ago, a new approach is needed.

**Approach:**

Using design thinking techniques, we hosted a full-day retreat for emergency medicine stakeholders in Hamilton and the surrounding region. Prior to the retreat we collected medico-legal data on emergency physicians in our region and performed a needs assessment survey. At the retreat, we had participants brainstorm ideas for CPD, generate archetypes for end-users, then generate solutions to the problems they had identified. These proposals were presented to the larger group for feedback and refinement.

**Evaluation:**

The Design Thinking Retreat generated five main pillars for action by our CPD team. 1) Simulation/procedural learning (staff simulation, procedural skills day, *in situ* simulation); 2) Asynchronous learning (website and podcast); 3) Synchronous learning (small group sessions for staff); 4) Community connectivity (online platform for collaboration and communication); and 5) Coaching & mentorship (focused coaching for specific practice improvement, improved onboarding for new staff).

**Reflection:**

These ideas have vastly increased engagement in CPD. Stakeholder consultation via design thinking may be a key approach for educators to use.

**Electronic supplementary material:**

The online version of this article (10.1007/s40037-020-00604-1) contains supplementary material, which is available to authorized users.

## Background and the need for innovation

As the clinical work environment changes and evolves, the needs of continuing professional development (CPD) evolve alongside. With changes in our clinical work environment (increasing technology, time constraints, increasing acuity/volume), and the ever-expanding amount of medical literature, these factors make it difficult to keep up with recent advances in the health sciences.

On top of the changing clinical environment, disruptive innovations are also altering how and what people learn [1]. The advent of social media (including the Free Open Access Medical (FOAM) education movement [2, 3]) has led to a drastic change in the way people access information via continuous access evidence updates (e.g. on blogs and in podcasts), threatening the traditional lecture-based conferencing models of continuing education in the health professions [1, 4–10]. Academically, new and existing faculty members are constantly being asked to do more with less. New faculty members (especially community faculty) can often find it difficult to establish their identity as teachers, scholars, and academics without proper support [11, 12], especially when they must learn new approaches such as competency-based medical education [13, 14].

Locally, CPD for emergency medicine in Hamilton has traditionally focused on regional rounds (lecture-based with a guest speaker) as well as a small annual conference (10:EM, a 10-minute emergency medicine conference). Noticing a declining attendance at both the rounds and the conference, we harnessed the power of design thinking [15, 16] and set forth to identify the CPD needs of emergency physicians in the area.

## Goal of innovation

The McMaster Emergency Medicine CPD team planned and executed a Design Thinking Retreat in September of 2018. We utilized the IDEO Design Thinking methodology to frame our process [16]. This method is utilized in the business sector, and refers to the processes (cognitive, strategic, and implementation) by which new products or workflows are designed. It has been adopted widely in many sectors, and is anchored in a constructivist epistemology – with heavy influences from the social sciences. This methodology uses alternating divergent (e.g. brainstorming) and convergent (e.g. decision-making) phases to help define problems and find solutions. There are five phases: Discovery, Interpretation, Ideation, Experimentation, and Prototyping. The goal was to gather a diverse group of faculty members together to rethink our regional needs for professional development. We aimed to have a cross section of academic and community practitioners, as well as other healthcare professionals to better understand the landscape of CPD for our diverse constituents.

## Steps taken for development and implementation of innovation

We assembled a one-day retreat full of a diverse group of educators from all across the region to investigate their local groups’ needs to develop a strategic plan for CPD in our region.

### Participants

We recruited 20 emergency physicians from a variety of sites in the area. Outside perspectives were introduced by two resident physicians and two paramedics who also attended, as well as a nurse who was a Director of Interprofessional Education at one of our affiliated hospitals.

### Organization of the day

A full-day retreat was planned and the agenda developed in accordance with the principles of the IDEO Design Thinking methodology [16]. In our one-day retreat, we harnessed the power of the first four phases of the process (Discovery, Interpretation, Ideation, and Experimentation), and launched into a follow-up period of 6 months wherein we prototyped various innovations developed during the day retreat. A description of what we did in each phase is listed below.

1. *Discovery phase*
  For the *Discovery* phase of our design thinking activity, we contacted the Canadian Medical Protective Association to gather information about what medico-legal complaints (legal, college and hospital complaints) emergency physicians in Ontario, Canada had experienced over a 5-year period. This report helped us to determine some of the unperceived needs of our target learners. For perceived learning needs, we also created a needs assessment survey and distributed it to local emergency physicians both within Hamilton and the surrounding regional hospitals affiliated with McMaster University. The survey was developed locally by our CPD team, piloted with non-participating colleagues from other institutions, and then distributed widely via our clinical and academic leadership. The response rate for the survey was 41% (80/195). Tab. 1 shows the results from the survey.

**Table 1:** Regional needs assessment survey responses

Questions	Responses (*n* = 80)
Years in practice	<5 years –35.4%
	5–10 years – 21.5%
	10–20 years – 16.5%
	>20 years – 25.3%
Practice sites	Community ED – 64.1%
	Tertiary care ED – 44.9%
	Urgent care – 38.5%
	Pediatric ED – 10.3%
Training path	CCFP-EM – 41.8%
	FRCPC-EM – 31.6%
	CCFP – 11.4%
	FRCPC-PEM – 7.6%
	Allied health – 3.9%
	ABEM – 3.8%
What are some roadblocks preventing you from further developing your career?	Lack of time 40% (*n* = 32)
	Lack of resource/funding 12.5% (*n* = 10)
	Lack of motivation/burnout 11.3% (*n* = 9)
	Lack of opportunities 10% (*n* = 8)
	Lack of mentorship 7.5% (*n* = 6)
What types of continuing professional education are you currently doing?	Conferences – 86.3%
	Podcasts – 77.6%
	Journal articles – 70.1%
	Rounds/lectures – 63.8%
	Workshops – 36.3%
	Simulation – 3.9%
What do you wish staff physician continuing professional education looked like?	Staff-only small group discussions
	‘Education responsive to our needs’
	Improved remote access/curated online content
	Staff simulation
	Procedural skills workshops

*ED* emergency department, *CCFP* Canadian College of Family Physicians, *EM* emergency medicine, *FRCPC* Fellow of the Royal College of Physicians of Canada, *PEM* Pediatric Emergency Medicine, *ABEM* American Board of Emergency Medicine
  The survey included demographic information about years in practice, training and practice type. Information about what type of CPD was currently being offered and how respondents felt about it was also elicited. Finally, attendees were given three questions prior to the retreat and were asked to interview three colleagues (one of each: early career, mid-career and late career) to obtain answers. The questions were as follows:
  What is the ‘coolest’ idea for CPD that you would want to see here?
  What would your ideal CPD program include?
  Is there any type of programming you feel like you can’t find elsewhere that you would want to see here?
2. *Interpretation phase*
  At the retreat all participants were briefed on the format for the day and then were provided with all the results of the needs assessment survey and Canadian Medical Protective Association report. They were asked to work in small groups, reviewing the information provided as well as the responses to their questions and then were instructed to brainstorm ideas. Participants were given specific sections of the various data sources (Fig. 1) and asked to break out into smaller groups. These groups were asked to engage in divergent thinking and brainstorm regarding the information about the data sources. They were tasked with isolating as many ideas about the barriers and enablers for our faculty members to attend CPD activities as possible. Each participant was asked to write each idea onto one sticky note and collect them within their team. After the teams completed brainstorming, this process had yielded hundreds of unique ideas. The participants were then asked to participate in a group activity to sort through all of the ideas, grouping them thematically in a real-time qualitative analysis.

**Fig. 1:**
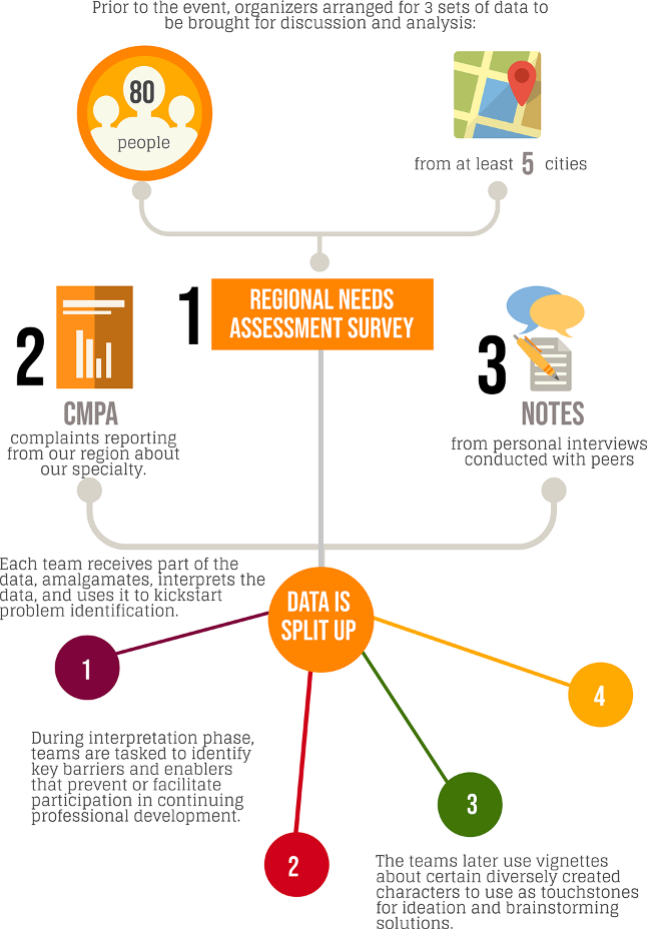
Depicting the process flow of information collection before our event, and distribution during the event
3. *Ideation phase*
  For the *Ideation* phase of the retreat, we once again divided our participants into small groups. Within these groups, we asked them to use the ideas we had generated and grouped previously to design an optimal suite of activities for some archetypal characters. Each of the archetypal characters were emergency physicians whom we anticipated might have fairly unique continuing education and developmental needs. The four characters we created were:
  - an early career clinical teacher;
  - an early-to-mid career physician returning from parental leave;
  - a late career physician;
  - an academic/tenure track physician with reduced clinical responsibilities.
  See the *online supplementary materials* (Appendix) for the handout we used to inspire discussions in these small groups.
  The groups were then asked to present their characters and their suggested continuing education plan to the group-at-large. Real-time notes were taken by the organizers (AC, TC) and an open discussion was facilitated to better understand the groups’ ideas and rationales as they came forth. Overarching, common themes from all four plans for the distinct archetypes were coalesced to establish an initial group of continuing education and development ideas for the groups to further refine.
4. *Experimentation phase*
  In the afternoon we moved on to the *Experimentation* phase, where we had the participants split into four groups to work on solutions for the CPD themes identified in the *Interpretation* phase. The groups generated and refined solutions separately as the facilitators rotated through the groups, challenging ideas and providing feedback.
  At the end of the *Experimentation* phase, the groups came back together and each one was tasked with pitching their main idea to the larger group. This allowed feedback and further refinement of the ideas generated. The group with the best pitch was awarded a prize. Further refinement and active piloting of ideas developed during this retreat were planned but not executed on this initial day, but have occurred since.

## Outcomes of the innovation

Based on program evaluation surveys (which were deemed exempt by our institutional review board), we received very positive feedback about the Design Thinking Retreat. The participants felt engaged by the process and that it allowed them to have their needs translated into actionable ideas. Participants also felt starting the process with data allowed us to explore the unperceived needs of the group.

As a result of the Design Thinking Retreat, our stakeholders generated five main pillars for our CPD Team to focus on over the next few years:

1. *Simulation/procedural learning*
  There has been considerable work done recently towards creating simulation opportunities for our trainees but not for staff physicians. This was an area the group felt needed change, and proposed three different options. The first was *in situ* simulation, which would have the dual benefit of being interprofessional and allowing us to identify latent safety threats in the emergency departments. The second was staff physician-only simulation sessions, which would allow staff to practice rare or challenging simulation cases in a safe environment where they felt comfortable making mistakes and receiving feedback. The third was a procedural skills day, where staff could come to practice procedures such as chest tubes, cricothyrotomies etc.
2. *Asynchronous learning*
  The nature of shift-work and busy schedules were identified as barriers to traditional CPD, so our stakeholders proposed more options for asynchronous learning. One aspect of this is a CPD website, which will help create a central access point not only for information about upcoming events, but also curated resources that staff can access in their own time. The group also discussed the benefits of a local podcast to highlight regional expertise and the hard work that our colleagues have been putting both in and out of the clinical setting.
3. *Synchronous learning*
  Although participants felt that the traditional rounds curriculum was valuable, they wanted a small group format where staff could comfortably discuss cases and the nuances of management. They proposed a staff-only journal club with invited speakers from other specialties where staff could get together, socialize, and discuss challenging cases and new evidence.
4. *Community connections*
  One of the barriers participants identified to CPD was a lack of information about resources available or events that were happening. Participants felt as though different sites were operating in silos, not collaborating on similar projects or working together to fix similar issues in their departments. In order to facilitate more collaboration and communication, they proposed using a private messaging/file sharing platform (in this case, Slack) to connect regional emergency physicians.
5. *One-on-one experiences (e.g. coaching & mentorship)*
  Finally, the group identified that a lack of feedback can inhibit professional growth after your pre-licensure structured training is over. They proposed creating a coaching program to help staff improve focused areas of their practice, such as clinical teaching, on shift efficiency or billing. They also identified that we were doing a poor job at on-boarding new hires to our academic or community teaching sites. They proposed a mentorship program that would help orient new staff to their clinical and academic roles and provide guidance as they built their careers.
  After the Retreat, the initial CPD team (AC, TC) needed to expand the working group to help achieve these goals. We recruited three additional staff physicians who had academic interests aligned with the five pillars of our new strategic plan. We voted a member into the position of CPD Director, and the other four remaining members were assigned portfolios as Associate Directors (for asynchronous learning, synchronous learning, coaching & mentorship, and simulation). Each Associate Director is responsible for his/her portfolio, with the Director helping and organizing the overall team. We also incorporated a 0.2 FTE administrative assistant into the team, who will help with logistics.
  Based on the outcome of our Design Thinking Retreat, we have created a larger, more representative CPD Team to meet the needs of our population of emergency physicians. We have tried to prioritize and create a timeline for the creation and implementation of the ideas generated during the retreat. A website and podcast (MacEmerg Podcast) have already been launched and are now in circulation. A small group, synchronous curriculum was launched in the spring of 2019 and we planned and delivered a procedural skills day in the winter of 2019. Simulation sessions for staff physicians are underway and have been receiving positive reviews.

## Critical reflection

While our stakeholder turnout for the retreat was excellent, it was by no means entirely representative of the group of physicians or health professionals in our region. The majority were from the four academic tertiary care centres and the results may be biased towards that population.

Some advice going forward for those who are interested in attempting this process:

- *Expand your data:* Consider the type of non-traditional CPD data you can gather from external sources. In our case, we used reports from a national malpractice database but others might find their hospital electronic health record a treasure trove of information.
- *Recruit a diverse group of stakeholders: *Try to involve as many people as possible that represent different segments of your potential CPD attendees (e.g. your market). We applied many filters to ensure we were able to recruit a diversity of individuals of varying genders, age, experience, and geographic location.
- *Don’t be afraid to ask your attendees to do some preparatory work: *Often for planning retreats attendees are asked to simply show up, but in doing so they are unprepared to offer insights that can be helpful. As such, we suggest that you find some reliable attendees within your stakeholder groups and ask them to prepare certain items. In our case, we asked individuals to conduct interviews with colleagues to get a better sense of what they liked or didn’t like about current CPD endeavours.
- *Plan to follow through: *The worst thing we could have done was to have a full-day retreat, get our stakeholders excited for change, and then not make any changes. Our CPD team was fully ready to launch into development based on the findings of the retreat, and this allowed us to earn some quick wins with our retreat attendees and our larger group of constituents. Aligned with Kotter’s framework for change [17], this certainly was helpful to our group in gaining trust and credibility from the larger group whom we serve.

One of the challenges of our design thinking technique is that it may be difficult to remain user-centred in our future developments in CPD. Our CPD executive have now all been through this activity; however, the ethos that emerges from the IDEO Design Thinking paradigm is one that has continued to resonate with our team going forward. We hope to conduct similar retreats at a later date, focusing on refining initiatives and increasing uptake of the great products we have prototyped and launched as a result of this initiative.

## Conclusions

Our planning retreat is a proof-of-concept that design thinking can be a powerful tool for gaining insights and developing strategy in education. This technique may be of use to other educators seeking to engage in stakeholder consultation in an efficient manner.

## Caption Electronic Supplementary Material

Contains materials for the handout the authors used to inspire small group discussions
