# Supplementary material for: Redesigning continuing professional development: Harnessing design thinking to go from needs assessment to mandate
Source: Perspect Med Educ. 2020 Aug 12;11(2):121–6. doi: 10.1007/s40037-020-00604-1 (PMC8941037; doi:10.1007/s40037-020-00604-1)
Supplement: Supplementary file 1 — Contains materials for the handout the authors used to inspire small group discussions [file 40037_2020_604_MOESM1_ESM.pdf]

Team A
Early career working in community

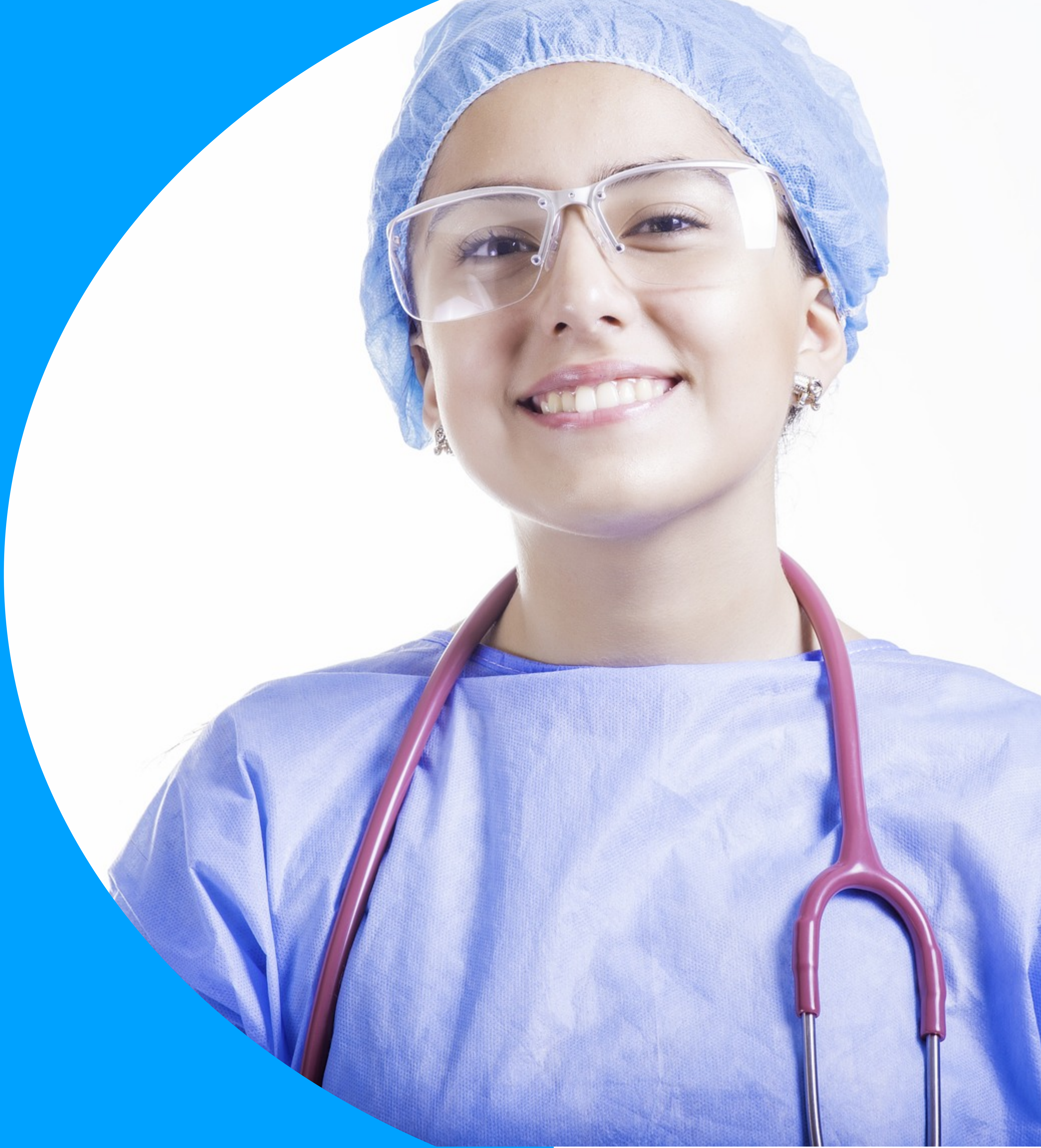

Photo from Pixabay.

Team B

5 years out just back from a 3 month mat leave

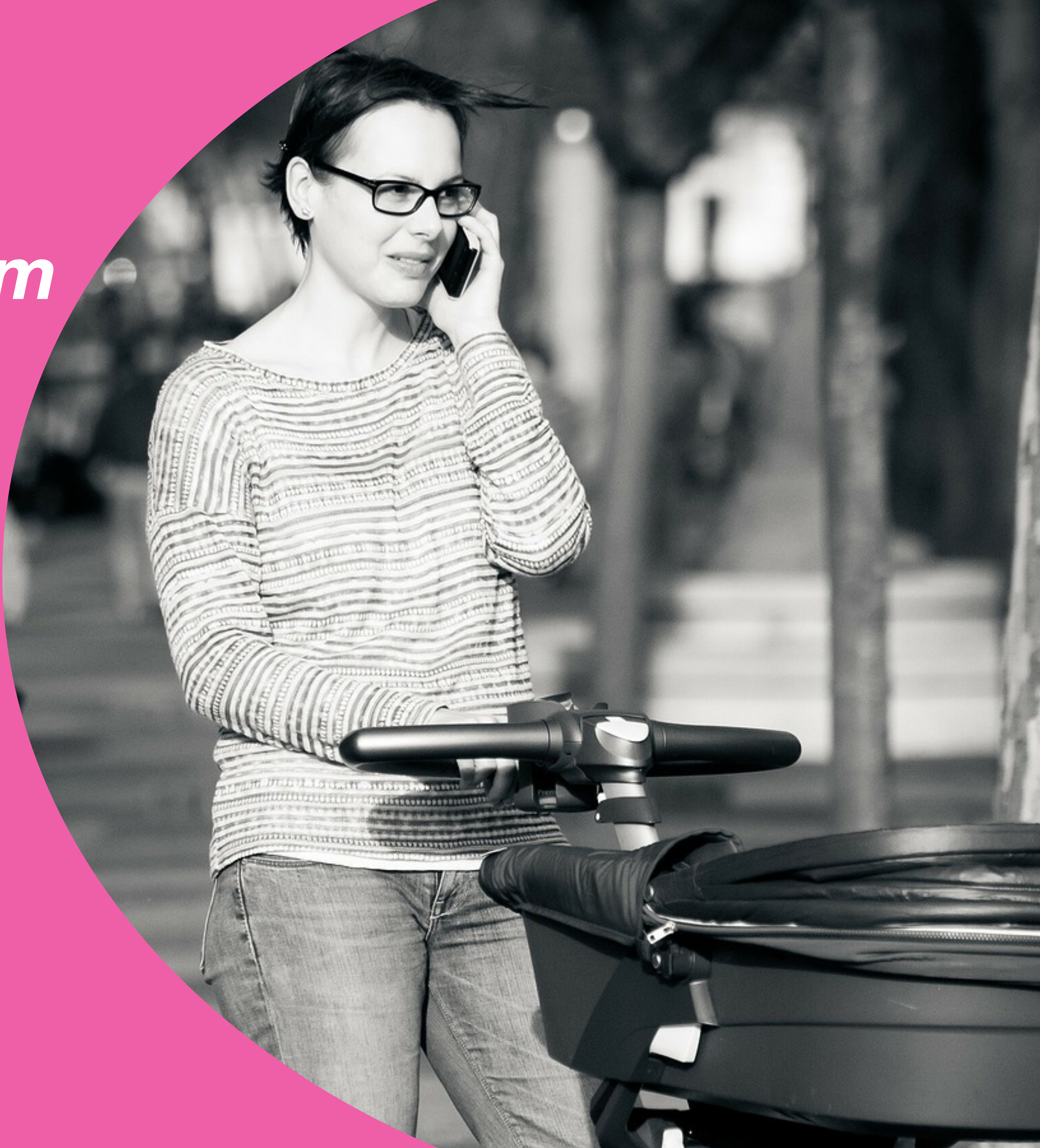

Photo from Pixabay.

Team C
Mid-career, academic position, working 6-shifts/mo

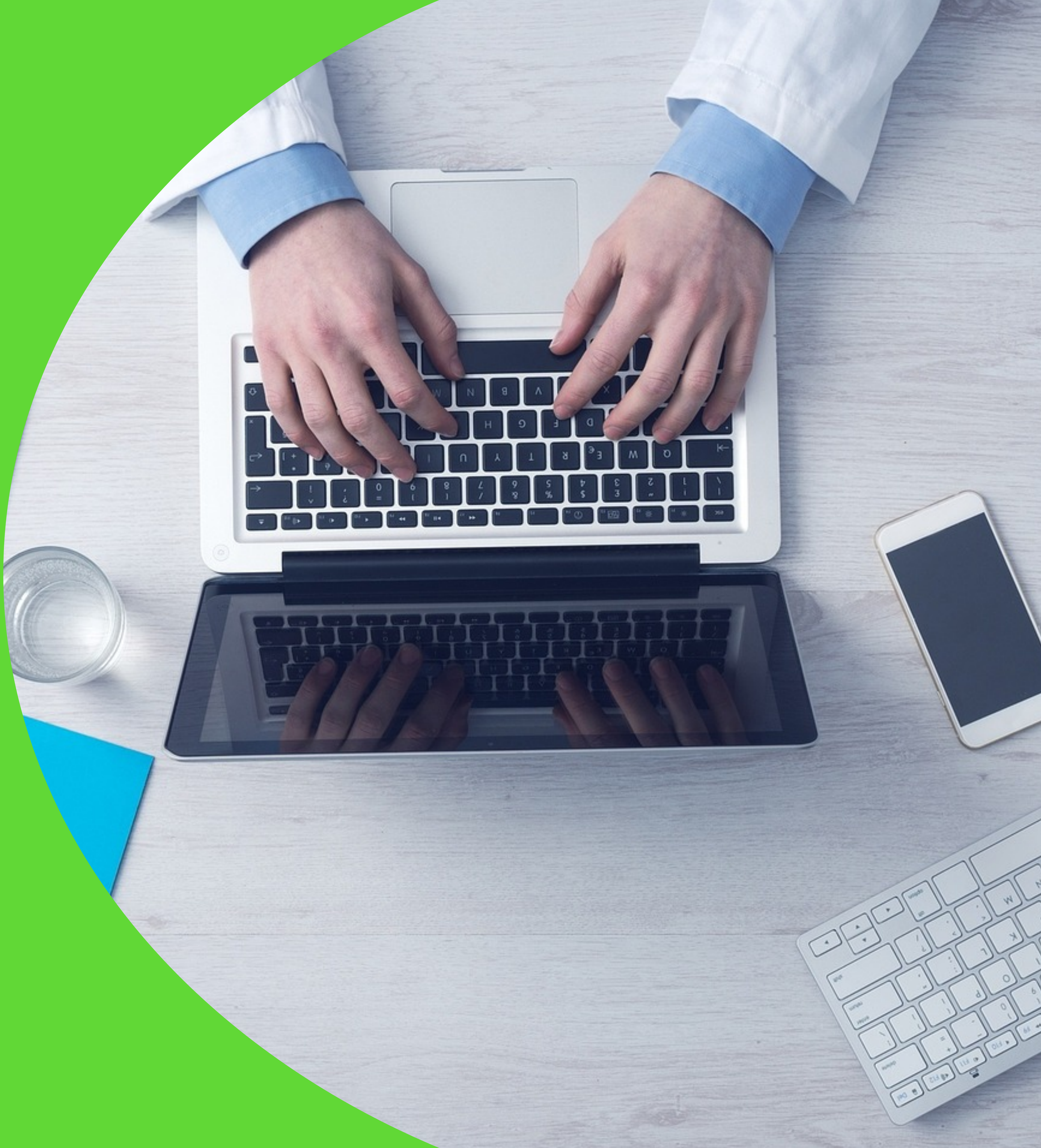

Photo from Pixabay.

Team D

Late-career, experienced clinician looking to expand their non-clinical role

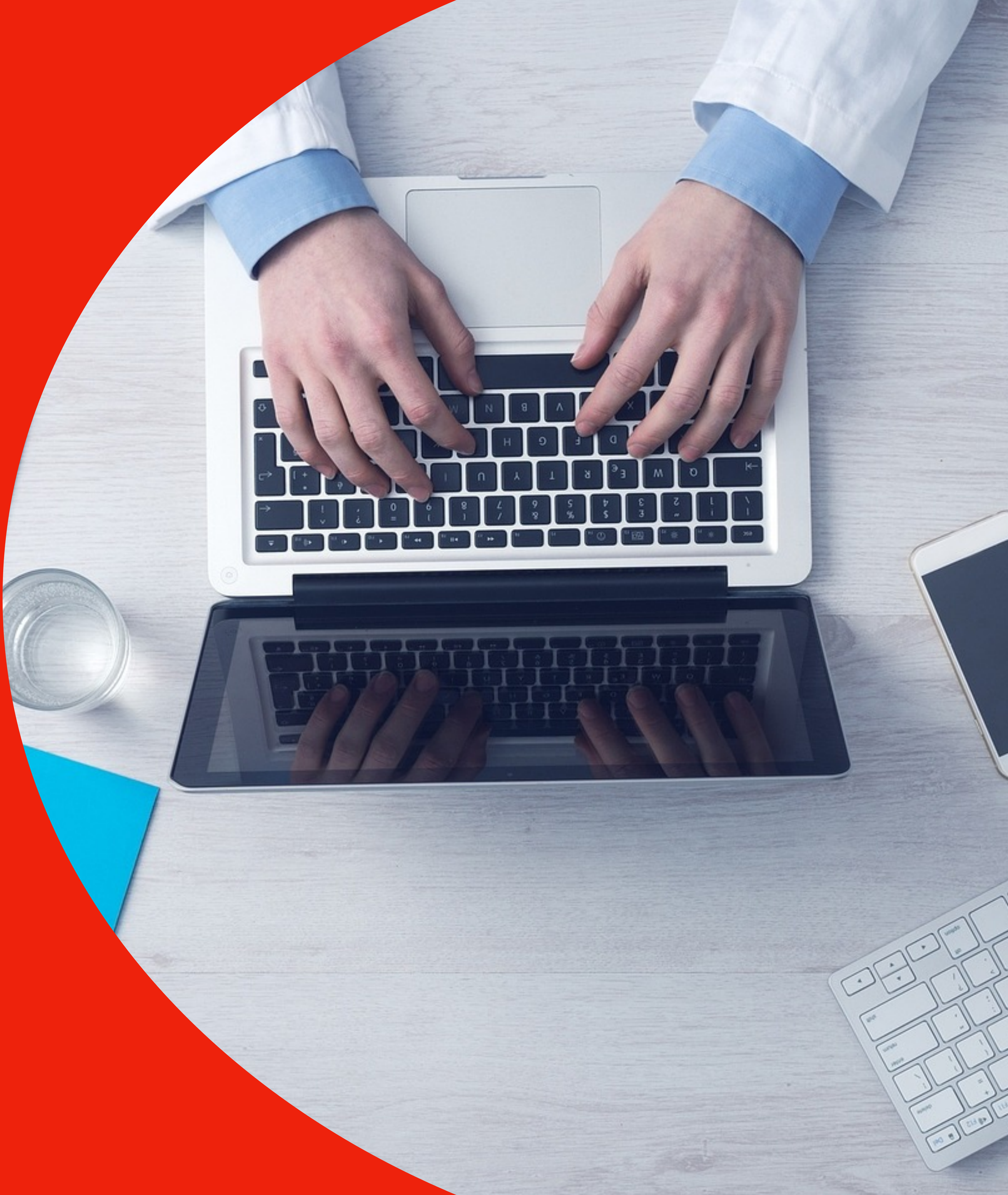

Photo from Pixabay.
